# Biliary Epithelial Senescence in Liver Disease: There Will Be SASP

**DOI:** 10.3389/fmolb.2021.803098

**Published:** 2021-12-21

**Authors:** Vik Meadows, Leonardo Baiocchi, Debjyoti Kundu, Keisaku Sato, Yessenia Fuentes, Chaodong Wu, Sanjukta Chakraborty, Shannon Glaser, Gianfranco Alpini, Lindsey Kennedy, Heather Francis

**Affiliations:** ^1^ Hepatology and Gastroenterology, Medicine, Indiana University, Indianapolis, IN, United States; ^2^ Hepatology Unit, University of Tor Vergata, Rome, Italy; ^3^ Clinical and Translational Sciences Institute, STEM GEHCS Program, Indiana University School of Medicine, Indianapolis, IN, United States; ^4^ Department of Nutrition, Texas A&M University, College Station, TX, United States; ^5^ Department of Medical Physiology, Texas A&M University College of Medicine, Bryan, TX, United States; ^6^ Richard L. Roudebush VA Medical Center, Indianapolis, IN, United States

**Keywords:** cholestasis, fatty liver, cell cycle arrest, bile duct, aging

## Abstract

Cellular senescence is a pathophysiological phenomenon in which proliferative cells enter cell cycle arrest following DNA damage and other stress signals. Natural, permanent DNA damage can occur after repetitive cell division; however, acute stress or other injuries can push cells into premature senescence and eventually a senescence-associated secretory phenotype (SASP). In recent years, there has been increased evidence for the role of premature senescence in disease progression including diabetes, cardiac diseases, and end-stage liver diseases including cholestasis. Liver size and function change with aging, and presumably with increasing cellular senescence, so it is important to understand the mechanisms by which cellular senescence affects the functional nature of the liver in health and disease. As well, cells in a SASP state secrete a multitude of inflammatory and pro-fibrogenic factors that modulate the microenvironment. Cellular SASP and the associated, secreted factors have been implicated in the progression of liver diseases, such as cholestatic injury that target the biliary epithelial cells (i.e., cholangiocytes) lining the bile ducts. Indeed, cholangiocyte senescence/SASP is proposed to be a driver of disease phenotypes in a variety of liver injuries. Within this review, we will discuss the impact of cholangiocyte senescence and SASP in the pathogenesis of cholestatic disorders.

## Introduction

Cholangiocytes, which are morphologically heterogenous, polarized cells lining the biliary epithelium (Han et al., 2013; Banales et al., 2019), have high absorptive/secretory functions and play a role in the *1*) modification of canalicular bile, *2*) paracrine communication with portal cells, and *3*) regulation of immune cell infiltration (Nathanson and Boyer, 1991; Chen et al., 2008; Banales et al., 2019). Cholangiocytes are the target of various liver diseases such as fatty liver diseases (non-alcoholic fatty liver disease [NAFLD], non-alcoholic steatohepatitis [NASH]), alcoholic liver disease (ALD) and chronic cholestatic liver diseases including primary biliary cholangitis (PBC), primary sclerosing cholangitis (PSC), biliary atresia, and cholangiocarcinoma. Biliary secretory functions regulate liver inflammation and fibrosis (by both autocrine and paracrine pathways) through secretion of cytokines and other factors which may contribute to liver damage (Kennedy et al., 2021; Kyritsi et al., 2021). Cellular senescence increases in cholangiocytes of PSC patients, likely contributing to disease progression (Tabibian et al., 2014a). Moreover, many factors secreted from cholangiocytes, such as interleukin (IL)-1β, IL-6, monocyte chemoattractant protein (MCP)-1, stem cell factor (SCF), transforming growth factor β1 (TGF-β1), and platelet derived growth factor (PDGF), are components of the senescence-associated secretory phenotype (SASP) (Rao and Jackson, 2016; Lopes-Paciencia et al., 2019; Blokland et al., 2020). Senescence and SASP secretion have gained considerable attention in studies of cholestatic liver disease progression, demonstrating a new role for senescent cholangiocytes in the pathogenesis of liver diseases (Knop et al., 1987; Wu et al., 2016).

## Cholangiocyte Cell Cycle Arrest and Progression

The liver is composed of two main types of epithelial cells: hepatocytes and biliary epithelial cells (i.e., cholangiocytes). In normal conditions, cholangiocytes represent less than 10% of total hepatic cellular mass in humans and 2–3% in rodents (Alpini et al., 1988); however, upon stimulation, they support bile acid (BA)-independent bile flow accounting for approximately 50% of total bile secretion (Kanno et al., 2000). Small and large cholangiocytes line small (<15 μm diameter) and large (≥15 μm diameter) bile ducts, respectively (Cheung et al., 2018). Heterogeneity in size closely relates to the difference in activity and functions of cholangiocytes. Large, cyclic adenosine monophosphate (cAMP)-dependent cholangiocytes represent the mature, hormone-dependent portion of biliary epithelium, while small Ca^2+^-dependent cholangiocytes are considered a typically quiescent population (Hall et al., 2017; Banales et al., 2019). Specifically, only small cholangiocytes proliferate when stimulated with a specific histamine receptor agonist (HRH1); however, small cholangiocytes can also acquire large cholangiocyte phenotypes after insult such as GABA treatment (Francis et al., 2008; Mancinelli et al., 2013). Secretory and proliferative activities of the cholangiocytes are finely tuned by several hormones, neuropeptides and angiogenic factors (Alvaro et al., 2007; Franchitto et al., 2013). For instance, secretin binding to the cholangiocyte-specific secretin receptor (SR, a G-protein coupled receptor) stimulates bicarbonate enriched choleresis, thereby increasing intracellular cAMP and activating the biliary cAMP/protein kinase A (PKA)-dependent cystic fibrosis transmembrane conductance regulator (CFTR) opening and subsequent anion exchange protein 2 (AE2) activation (Glaser et al., 2009; Mancinelli et al., 2013; Wu et al., 2020). Fluctuations in intracellular levels of cAMP, and its role as a second messenger, are important in the biliary epithelia not only for secretory activities, but also for proliferative processes (Baiocchi et al., 2021). Large cholangiocyte proliferation is tightly regulated by cAMP-dependent PKA/proto-oncogene tyrosine-protein kinase Src (Src)/mitogen activated protein kinase kinase (MEK)/extracellular signal-regulated protein kinases 1/2 (ERK1/2) signaling axis (Francis et al., 2004). Three proliferative modalities have been identified in experiments in normal and pathological biliary conditions (Alvaro et al., 2000). Type I (typical proliferation) is represented by an inordinate hyperplastic growth of bile ducts and is observed in the cholestatic injury model of bile duct ligation (BDL) in rodents (Alvaro et al., 2007; Glaser et al., 2009). Conversely, Type II (atypical proliferation) spreads outside the portal space, with irregular truncated ducts and is associated with chronic cholestatic diseases or severe injuries (Franchitto et al., 2013). Finally, Type III proliferation is characterized by expansion of the “oval” cells (i.e., hepatic progenitor cells, located in the Canals of Hering) and is characteristic of BDL and 3,5-diethoxycarbonyl-1,4-dihydrocollidine (DDC) diet in rodent models and in human PBC (Olynyk et al., 1998; Carpino et al., 2018; Fragoulis et al., 2019). While each type of proliferative modality is separate, many cholestatic models, such as BDL, may exhibit multiple subtypes during disease progression.

On the opposite side of active eukaryotic cell division and growth stands cell cycle arrest (Toettcher et al., 2009). This molecular process promptly activates during conditions of cellular stress and DNA damage, thus preventing cell damage/repair and ceasing normal replicative activities. When cell repair cannot accommodate increased DNA damage, cell cycle arrest may evolve toward a programmed cell death route (Wiman and Zhivotovsky, 2017), such as autophagy (Glick et al., 2010), necroptosis (Khoury et al., 2020) and apoptosis (Fleisher, 1997). Homeostatic regulation of growth by apoptosis has been confirmed in biliary epithelia (Bhathal and Gall, 1985). In fact, proliferative insult to rat cholangiocytes (either BDL or α-naphthyl isothiocyanate administration) has been shown to determine an apoptosis-dependent restoration of normal bile duct mass (Lesage et al., 2001). From these and other findings, apoptosis deregulation has been suggested to play an important role in determining and maintaining ductopenic cholangiopathies, such as human PBC or PSC (Celli and Que, 1998). However, in recent years, cell cycle arrest has gained attention for the possibility to give rise to a senescent cellular phenotype. This seems associated with tissue aging, as well as acute damage from pathological conditions, including those affecting the biliary system since senescent cells are generally anti-apoptotic (Childs et al., 2014).

## Aging and Senescence

Aging is a complex process. Several factors contribute to this progression including genetic, mitochondrial, peptidic and paracrine components (López-Otín et al., 2013); however, at the cellular level a specific phenotype has been consistently identified in aged tissue: the senescent cell (Campisi, 2013). This phenotype is characterized by an irreversible interruption of replicative phases possibly to prevent oncogenic proliferation after cellular injury (Sager, 1991). Two other specific features belong to a senescent cell: *1*) lack of progression toward apoptosis; and *2*) development of a SASP (Coppé et al., 2008). Following the induction of SASP, senescent cells are considered to contribute to some degenerative diseases. Experiments in transgenic mice with deletion of senescent cells, demonstrated improved outcomes of neurologic, vascular and musculoskeletal degenerative processes (Campisi et al., 2019). It is important to underscore that the complex and heterogeneous SASP paracrine stimuli negatively or positively affect the microenvironment depending upon the pathophysiological setting. SASP may contribute to the prevention of cancer or even tissue repair (Campisi, 2013). In this perspective, the strong activity of SASP in wound healing seems in conflict with the reduced regenerative capability of aged tissue.

## Senescence in Cholangiopathies

### Primary Biliary Cholangitis

PBC is an autoimmune chronic liver disease characterized by cholestasis and ductopenia of the interlobular bile ducts (Onofrio et al., 2019). PBC primarily affects middle-aged women who may present with abdominal discomfort, pruritus, fatigue or no symptoms, complicating its complex etiology (Onofrio et al., 2019). Diagnostic criteria for PBC include positive serum anti-mitochondrial antibodies (AMA) and chronic (∼24 weeks) abnormal ALP levels or comparable liver histology analysis (Onofrio et al., 2019). Recent studies have advanced diagnosis and treatment options for PBC patients, but therapeutics targeting senescence have not been evaluated in the clinical settings (Onofrio et al., 2019). Due to increased bile duct senescence in PBC patients, understanding biliary senescence/SASP development remains a novel strategy to understand disease progression.

Many correlative studies have evaluated the expression of senescence and SASP in human PBC samples (Sasaki et al., 2005; Sasaki et al., 2006; Sasaki et al., 2008), but mechanistic studies are scarce. Telomere shortening has been noted in the small ducts of PBC patients and is found in conjunction with expression of p16 and p21 (Sasaki et al., 2008). Another study has shown that small ducts of PBC patients highly expressed senescent markers, and this was associated with the development of non-suppurative cholangitis and the portal infiltration of inflammatory cells (Sasaki et al., 2005). Indeed, enhanced biliary senescence is a driver of worsening disease in PBC, but the cause of senescence induction is complicated. A potential cause for this increased senescence includes enhanced oxidative stress. *In vitro* work demonstrated that biliary senescence may be driven by enhanced oxidative stress as determined by H_2_O_2_ and nitric oxide stimulation (Sasaki et al., 2005). This theory is supported by work that found increased endoplasmic reticulum (ER) stress, which is associated with oxidative stress, in the small ducts of patients with PBC, and the *in vitro* induction of ER stress promoted senescence in cultured cholangiocytes (Sasaki et al., 2015). Enhanced cell stress is an understandable component of biliary senescence considering the large degree of immune cell-induced damage to the cholangiocytes that occurs during PBC. Additionally, understanding the impact of enhanced biliary senescence during PBC pathogenesis is relevant considering high-risk PBC patients, identified by impaired ursodeoxycholic acid (UDCA) response, have increased p21 gene and protein expression when compared to low-risk PBC patients (Hardie et al., 2016). Recent work characterized the serum proteome of PBC patients and found that patients with attenuated response to UDCA had enhanced serum levels of chemokines associated with senescence, which the authors postulate to be released from senescent cholangiocytes (Barron-Millar et al., 2021). Specifically, the serum levels of C-X-C motif chemokine ligand 11 (CXCL11) and chemokine ligand 20 (CCL20) were significantly inversely correlated with UDCA response (Barron-Millar et al., 2021). This work underlines the importance of senescence in PBC pathogenesis, but also identifies new prognostic factors to be utilized for treatment strategies (Barron-Millar et al., 2021). Additionally, others demonstrated that inducing senescence in cholangiocytes *via* oxidative stress, DNA damage and serum deprivation *in vitro* leads to a subsequent increase in secretion of various chemokines (Sasaki et al., 2010a). These senescent cholangiocytes enhanced macrophage recruitment, which was blocked using neutralizing antibodies against CCL2 and fractalkine (i.e., chemokines) (Sasaki et al., 2010a). Of relevance, CCL2 and fractalkine expression was increased in inflamed bile ducts in human PBC samples (Sasaki et al., 2010a). Moreover, senescent small ducts in PBC also express genes associated with inflammation and immune response (Sasaki et al., 2021). This work further highlights that biliary senescence induces a pro-inflammatory secretome within damaged cholangiocytes, which promotes inflammation and the recruitment of various inflammatory cells. These mechanisms are obvious drivers of a worsening prognosis, considering the association of biliary senescence with UDCA response, and so it is imperative we define these mechanisms to understand disease progression.

Aside from oxidative stress, autophagy has been implicated as a driver of cholangiocyte senescence during PBC. Expression of autophagy markers are increased in damaged small ducts in PBC patients and are co-localized with senescence marker expression (Sasaki et al., 2010b). In cultured cholangiocytes, inhibition of autophagy blocked senescence (Sasaki et al., 2010b). Autophagy markers were expressed in the ductular reactive cells of both early- and late-stage PBC patients, whereas senescent markers were predominantly found in late-stage PBC, suggesting that autophagy precedes senescence development (Sasaki et al., 2012a). Similarly, p62 sequestrome-1 (p62, involved in autophagy) aggregates are found in inflamed small ducts in PBC and are co-expressed with obligate autophagy and senescent markers (Sasaki et al., 2012b). Knockdown of p62 reduced autophagy and senescence in cultured cholangiocytes (Sasaki et al., 2012b), lending reason to a potential link between autophagy and biliary senescence in PBC initiation and progression. These studies suggest a mechanism preceding biliary senescence in PBC, which can be used as a target to block the development of biliary senescence and subsequent liver injury. *In vitro*, treatment with hydrophobic BAs reduced AE2 expression, and enhanced oxidative stress and senescence in human cholangiocytes, showing that the hepatic microenvironment may initiate biliary oxidative stress and senescence. (Hisamoto et al., 2016). Since loss of the bicarbonate umbrella, specifically through reduced AE2 expression, is a hallmark of PBC, this data is quite compelling (Arenas et al., 2019; Banales et al., 2012). In human samples, decreased expression of AE2 is correlated with increased bile duct senescence during PBC (Sasaki et al., 2018a), thus identifying that the bicarbonate umbrella may be an important component of PBC progression *via* increased senescence. As well, these studies link loss of the bicarbonate umbrella, a cholangiocyte-specific protective mechanism, with biliary senescence which is a novel and new finding. Interestingly, both the bicarbonate umbrella and SASP factor release can be utilized to identify unique biliary signatures in PBC, which may point to new mechanisms or markers. Upstream of AE2 activity is secretin/SR signaling, that is a determinant modulating the bicarbonate umbrella and bile flow (Jones et al., 2015). When looking at early-stage PBC, one study found enhanced secretin/SR expression in bile ducts of human samples and the dominant-negative TGF-β receptor II mouse model of PBC (Kennedy et al., 2019). In this murine model of early-stage PBC, treatment with a SR antagonist (Sec 5–27) reduced SR activation and subsequently decreased TGF-β1 expression, biliary senescence, and liver fibrosis (Kennedy et al., 2019). This study found elevated secretin/SR axis and subsequent TGF-β1 secretion and TGF-βR1 expression in human early-stage PBC samples compared to healthy control, insinuating a role for secretin/SR in PBC development and subsequent senescence (Kennedy et al., 2019). This work describes an upstream pathway regulating biliary senescence/SASP during PBC, and this mechanism may potentially be targeted for therapeutic use.

Underscoring our previous comments, direct targeting of senescence or other factors driving senescence may be therapeutic for PBC treatment, and this has been tested in experimental models. Expression of senescent markers (p16 and p21) and the anti-apoptotic marker, B cell lymphoma-extra-large (Bcl-xL), are enhanced in the small ducts of patients with PBC (Sasaki et al., 2020). Specifically, senescent cholangiocytes were found within the ductular reactions and corresponded with stage, hepatitis activity and inadequate response to UDCA (Sasaki et al., 2020). Lastly, the authors induced senescence in murine cholangiocytes *in vitro* and found that treatment with senolytics (AA-1331852, Navitoclax, Dasatinib and Dasatinib with Quercetin) induced apoptosis and effectively cleared senescent cholangiocytes (Sasaki et al., 2020). The polycomb group gene, Bmi1 downregulates p16 expression, and reduced expression of Bmi1 in the small ducts of PBC, and *in vitro* oxidative stress was able to repress Bmi1 expression in cultured cholangiocytes (Sasaki et al., 2006). Together, these findings confirm an associated theme of oxidative stress promoting senescence in PBC. Antioxidant therapies may remedy PBC-associated injury through reduced biliary senescence; however, it is imperative that we delineate the role of biliary senescence in PBC pathogenesis to define additional therapeutic options for patients. This section has highlighted the potential for antioxidant and senolytic therapies for the treatment of PBC.

### Primary Sclerosing Cholangitis

PSC is an idiopathic chronic cholangiopathy characterized by increased hepatic inflammation, bridging fibrosis and progressive cholestasis (Lazaridis and LaRusso, 2015). Much like PBC, senescent cholangiocytes have been implicated in PSC progression and exacerbation of hepatic damage through paracrine secretion of inflammatory and fibrotic factors (Lazaridis and LaRusso, 2015). Therapeutic options for PSC patients are limited, with liver transplantation serving as the sole therapeutic option to improve long-term survival (Rawla and Samant, 2021). A common feature in PSC is reactive cholangiocytes, which may exhibit proliferative or senescent marker expression and increased inflammatory and fibrotic factor secretion (Lazaridis and LaRusso, 2015). The role of biliary senescence in PSC has been of rising interesting, but its exact impact on disease progression remains undefined.

Early work using primary cholangiocytes isolated from PSC patients found that these cholangiocytes highly express the senescence markers, p16 and γH2A.X, compared to normal cholangiocytes (Tabibian et al., 2014a). Similarly, another study found that cholangiocytes isolated from PSC patients and maintained in primary culture have a lower proliferative rate and high expression of SA-β-galactosidase when compared to normal cholangiocytes (Tabibian et al., 2014b). PSC cholangiocytes were also found to have increased secretion of SASP components (IL-6, IL-8, CCL2, PAI-1) compared to normal cholangiocytes, and these SASP factors induced senescence in bystander cholangiocytes as indicated by *in vitro* co-culture systems (Tabibian et al., 2014a). In this study, the induction of biliary senescence was driven by neuroblastoma RAS viral oncogene homolog (N-Ras) activation in cholangiocytes (Tabibian et al., 2014a). Others have found that cholangiocytes of PSC patients have increased expression of senescence factors as indicated by staining in liver sections (Ferreira-Gonzalez et al., 2018). Together, all of these studies suggest that biliary senescence is a key component of PSC, and similar to PBC it can confer a worsening phenotype through modulation of nearby cells. A novel murine model of inducible cholangiocyte senescence has found that upregulation of p53, through knockout of mouse double minute 2 proto-oncogene (Mdm2), leads to increased p16, p21 and γH2AX in bile ducts (Ferreira-Gonzalez et al., 2018). This work found that senescent cholangiocytes have elevated secretion of SASP factors with paracrine consequences such as increased hepatocyte senescence, exacerbated hepatic fibrosis and decreased liver regeneration (Ferreira-Gonzalez et al., 2018). Interestingly, the induction of biliary senescence in normal mice promoted portal macrophage infiltration and peribiliary fibrosis which aggravated these phenotypes when combined with DDC feeding to induce cholestasis (Ferreira-Gonzalez et al., 2018). This work demonstrates that biliary senescence alone can promote phenotypes associated with PSC, and this conclusion brings forth the question on the ability of cholangiocyte senescence to be a component of PSC etiology. In an *in vitro* model, cholangiocyte organoids (cholangoids) made from normal patient cholangiocytes that are exposed to H_2_O_2_ (to induce senescence, termed NHC-sen) display senescence and SASP characteristics. Further, NHC-sen cholangoids, as well as PSC cholangoids enhance macrophage recruitment compared to control (Guicciardi et al., 2018). Interestingly, isolated cholangiocytes from PSC patients have increased expression of genes associated with cell cycle arrest and senescence, as indicated by RNA-seq, and senescent cholangiocytes formed cholangoids of a smaller size that also lacked a lumen when compared to normal cholangiocytes (Jalan-Sakrikar et al., 2021). This work is supported by other findings showing that NHC-sen cells promote the proliferation of healthy cholangiocytes and monocyte migration, which was also found with plasma EVs from multidrug resistant cassette 2 knock out (*Mdr2* ^−/−^) mice, a genetic murine model of PSC (Al Suraih et al., 2020). The identification of increased biliary senescence in PSC is worth intensive evaluation since it is correlated with DR, fibrosis staging and other markers of severe disease in PSC patients (Cazzagon et al., 2021). Together, the above work demonstrate that biliary senescence can induce senescence of nearby cholangiocytes and drive hepatic damage associated with cholangiopathies, such as PSC.

A feature of senescent cholangiocytes is a pro-inflammatory and pro-fibrotic secretome. One study found that biliary-derived TGF-β1 promoted paracrine cholangiocyte senescence (Ferreira-Gonzalez et al., 2018) and it has been shown that TGF-β1 blocks cell cycle progression by enhancing the transcription of senescent factors such as cyclin-dependent kinase inhibitors, p21 and p27 (Datto et al., 1995). Indeed, others have found TGF-β1 promotes cholangiocyte senescence and subsequent liver fibrosis through autocrine and paracrine signaling (Wu et al., 2016). Increased biliary TGF-β1 synthesis and secretion was found to be downstream of the secretin/SR pathway, only expressed by cholangiocytes in the liver (Wu et al., 2016) thereby demonstrating a cholangiocyte-specific mechanism regulating biliary senescence in cholangiopathies. Additionally, the inhibition of secretin/SR signaling using Sec 5–27 or SR^−/−^ mice reduced biliary senescence, TGF-β1 levels and liver fibrosis in models of PSC (BDL and *Mdr2* ^−/−^ mice) (Wu et al., 2016; Zhou et al., 2018). It is apparent that cholangiocyte-derived TGF-β1 is an important factor mediating biliary (autocrine) and liver (paracrine) damage during PSC. Aside from cholangiocytes, mast cells secrete TGF-β1 and induce biliary senescence and other markers of cholestasis (Kyritsi et al., 2021), which is an important feature considering that mast cell migration to portal areas is a key feature and damaging component of cholestasis (Wilcox et al., 1986; Tsuneyama et al., 1999; Jones et al., 2016). Mast cell farnesoid X receptor (FXR) signaling promotes biliary senescence and associated biliary and liver damage in murine models of PSC (Meadows et al., 2021). Mast cell-derived components lend to worsening phenotypes in PSC and understanding if they can be targeted to mediate damage is a topic of research currently. Stem cell factor (SCF) is another cholangiocyte secretory component, and SASP factor, found to promote senescence in models of PSC (Meadows et al., 2019). It was demonstrated that SCF Vivo-Morpholino treatment (to reduce SCF expression) significantly decreased biliary senescence, as well as DR and liver fibrosis in *Mdr2* ^−/−^ mice (Meadows et al., 2019). In addition, this study demonstrated enhanced serum SCF and biliary SCF expression in human PSC compared to control samples (Meadows et al., 2019). This finding is corroborated by a correlative study demonstrating aberrant biliary SCF expression in human PSC samples (Tsuneyama et al., 1999). Interestingly, cholangiocyte SCF expression correlated with increased portal infiltration of cKit (SCF receptor) positive mast cells in human PSC (Tsuneyama et al., 1999), and SCF Vivo-Morpholino reduced hepatic mast cell presence in *Mdr2* ^−/−^ mice (Meadows et al., 2019), insinuating that biliary senescence mediates mast cell number and activity during PSC. This work further lends to the hypothesis that cholangiocyte secreted SASP factors can modulate the microenvironment and promote damage through the recruitment of immune cells, including mast cells.

Other signaling factors have been implicated in PSC-associated biliary senescence, such as histamine signaling, as demonstrated when *Mdr2* ^−/−^ mice were treated with a Vivo-Morpholino targeting the H2 histamine receptor (H2HR) inhibited biliary senescence, liver inflammation and fibrogenesis (Kennedy et al., 2020). Interestingly, this study determined that H2HR inhibition selectively targets large cholangiocyte senescence through downregulation of TGF-β1 expression (Kennedy et al., 2020), which again underlines the significance of TGF-β1 signaling in cholangiocytes during cholestatic injury. Biliary heterogeneity is an important factor influencing PSC damage. Classic PSC can affect both small and large bile ducts while small-duct PSC demonstrates fibrosis and inflammation of small ducts alone (Rawla and Samant, 2021). Understanding heterogeneity of PSC senescence is important since FoxA2, a definitive endoderm marker and transcription factor, is reduced in human PSC (McDaniel et al., 2017). Previous work has also demonstrated that this factor is predominantly expressed in small mouse cholangiocytes and liver progenitor cells *in vitro* and transplant of cultured small mouse cholangiocytes enhances FoxA2 expression, reduces biliary senescence and liver fibrosis in BDL and *Mdr2* ^−/−^ mice (McDaniel et al., 2017). A preferential increase in large bile duct mass has been noted in *Mdr2* ^*−/−*^ mice, demonstrating that this subpopulation may be more prone to injury and thus senescent damage (Kennedy et al., 2018a). In this regard, biliary heterogeneity may be an important aspect of biliary senescence, but more studies are warranted. It is imperative that we better define biliary heterogeneity in humans, specifically in the context of disease, to understand if these pathways identified in mouse models can hold true in the clinical setting.

Aging, in the context of disease, can work hand-in-hand with senescence to perturb liver injury (Kundu et al., 2020). Aged mice show increased expression of microRNAs (miRs) associated with aging processes (miR-1a, miR-20a, miR-30e), and increased expression of these miRs correlated with increased twinfilin-1 (twf-1) levels (Maroni et al., 2019). Interestingly, twf-1 expression increased in the DDC feeding model of cholestasis and in human PSC samples, and *Twf-1* ^*−/−*^ mice subjected to DDC had reduced biliary senescence and SASP (Maroni et al., 2019). Additionally, senescence-accelerated mice (SAMP8) had increased twf-1 expression and subsequent biliary senescence/SASP (Maroni et al., 2019). However, little work on the inappropriate induction of aging processes in cholestasis, including PSC, has not been evaluated thus far. Identifying signals inducing aging pathways cholangiocytes will be an important finding for PSC studies. Interestingly, the microbiota has been implicated in biliary senescence, as well (Tabibian et al., 2016). *Mdr2* ^−/−^ mice housed in a germ-free facility had exacerbated biliary senescence/SASP, associated DR and liver fibrosis (Tabibian et al., 2016). This is not surprising considering the gut-liver axis is vital for physiological function. This work also demonstrates that we can target factors outside of the liver in an effort to reduce biliary senescence during PSC, and this can potentially be applied to other cholestatic injuries as well.

Since cholangiocyte senescence and SASP are related to PSC-associated biliary and liver damage, it is of interest to evaluate if blocking biliary senescence/SASP may ameliorate damage. One study treated *Mdr2* ^−/−^ mice with a p16 Vivo-Morpholino and found that this treatment reduced p16 expression and senescence, TGF-β1 expression and biliary secretion of SASP components (Kyritsi et al., 2020). Furthermore, p16 Vivo-Morpholino reduced DR and portal fibrosis in *Mdr2* ^−/−^ mice compared to controls, which was shown to be linked to reduced miR-34a/Sirtuin-1 (SIRT1) signaling (Kyritsi et al., 2020). This is relevant considering others have found SIRT1 activity to promote cholangiocyte senescence in a model of obstructive cholestasis (Jia et al., 2021). One study generated an *Mdr2* ^−/−^/p16^−/−^ mouse, as well an *Mdr2* ^−/−^ mouse line crossed with the p16Ink4a apoptosis, through targeted activation of caspase (INK-ATTAC) mouse capable of selective clearance of p16-expressing cells, and found that both of these models had reduced biliary senescence and SASP, and subsequent decreased inflammation and portal fibrosis (Alsuraih et al., 2021). Additionally, the authors found that fisetin, a flavonoid that acts as a senolytic, induced similar phenotypic changes in *Mdr2* ^−/−^ mice, and *in vitro* selectively targeted senescent cholangiocytes (Alsuraih et al., 2021). These studies are in parallel with those in PBC by demonstrating that blocking biliary senescence may be therapeutic for the treatment of PSC. Lastly, it is known that senescent cells are resistant to apoptotic clearance as demonstrated by enhanced Bcl-xL expression in senescent cholangiocytes, as one study displayed the protective mechanism of biliary apoptosis in a model of PSC (Moncsek et al., 2018). Specifically, the clearance of senescent cholangiocytes by Bcl-xL inhibitor treatment reduced liver fibrosis and inflammatory marker expression (Moncsek et al., 2018). These studies establish the powerful impact of targeting biliary senescence for the treatment of PSC and the gaps in knowledge to be filled in with future studies.

### Biliary Atresia

Biliary atresia is a devastating pediatric cholestatic and fibrogenic liver disease with a multifactorial etiology and unknown molecular mechanism of pathogenesis (Bezerra et al., 2018; Kerola et al., 2019). Early intervention with Kasai hepatic portoenterostomy, to restore bile flow, and eventual liver transplantation increases survival of biliary atresia patients (Bezerra et al., 2018). Due to its complex pathology, a major challenge with biliary atresia treatment includes therapeutic target identification for early diagnosis. Like in adult cholestatic liver diseases, biliary and hepatic progenitor cells (HPC) senescence may exacerbate liver damage; however, research on senescence/SASP factors in biliary atresia remains severely understudied (Sasaki et al., 2018b; Wu et al., 2018; Xiao et al., 2019; Winkler et al., 2021). In the following section we highlight key studies implicating biliary senescence, and SASP factors, as contributors to biliary atresia development.

Biliary atresia patients show reduced telomere length in hepatic tissues, demonstrating telomere length negatively correlates with pediatric end-stage liver disease score (Sanada et al., 2012). The stress of biliary atresia can be measured outside of the liver, implicating far reaching effects of premature liver dysfunction (Udomsinprasert et al., 2015). Peripheral leukocytes in biliary atresia patients had reduced telomere length compared to healthy control (Udomsinprasert et al., 2015). This work found that leukocyte telomere length shortened with biliary atresia progression and had positive correlation with hepatic telomere length (Udomsinprasert et al., 2015). Interestingly, this study found that in two sets of identical twins, the twin with biliary atresia presented with reduced telomere length in hepatic tissue compared with the healthy twin (Udomsinprasert et al., 2015). These data allow us to postulate that stress from exhaustive proliferation of cholangiocytes may result in biliary senescence and diminishing immune intervention in disease progression in biliary atresia patients. Genomic instability caused by shortened telomere length initiates apoptosis and senescence and provides a therapeutic avenue for biliary atresia patients upstream of senescence (López-Otín et al., 2013). Previous work demonstrated that cholangiocytes from the adeno-associated virus (AAV) murine model of biliary atresia, expressed major histocompatibility complex (MHC) I and II, but did not serve as antigen presenting cells. Instead, these cholangiocytes modulated immune response by secretion of pro-inflammatory cytokines and chemokines (tumor necrosis factor α [TNFα] and TGF-β) (Barnes et al., 2009). This study identifies that biliary immunobiology may be an important component of biliary atresia progression through the release of inflammatory mediators. While this study does not discuss biliary immune regulation in the context of senescence/SASP, it does point to a hypothesis whereby senescent cholangiocytes may have dysregulated expression of MHC components or other immune signaling factors, or vice versa, that contribute to biliary atresia.

Senescent cholangiocyte present with enhanced p16 and p21 expression, particular in DR, in PBC and PSC; however, the role of biliary senescence in pediatric cholangiopathies like biliary atresia is limited (Sasaki et al., 2018b). Biliary and HPC p16 expression was found to be elevated in biliary atresia explant livers, indicating senescence as a component of biliary atresia pathogenesis (Sasaki et al., 2018b). Biliary atresia patients undergoing Kasai portoenterostomy have increased neural cell adhesion molecule (NCAM) positive DR cells, a stemness marker expressed by HPCs in the liver, with minimal NCAM-positive bile ducts (Sasaki et al., 2018b). However, at the time of liver transplantation, these patients had elevated NCAM expression in DR and bile duct cells, implicating differentiation during disease progression. This study also found a positive correlation between bile duct p21 expression and bile duct loss in biliary atresia patients, insinuating that the NCAM-positive DR cells (suspected to be HPCs) could be attempting to resolve bile duct loss in biliary atresia patients by differentiating into bile duct epithelial cells (Sasaki et al., 2018b). This complex study discusses HPC proliferation and differentiation into cholangiocytes in the context of DR and cholangiocytes senescence, but the signaling mechanisms are complicated and additional work is necessary.

A murine model of inducible cholangiocyte senescence shows that senescent cholangiocytes have elevated secretion of SASP factors with paracrine consequences such as increased hepatocyte senescence, exacerbated hepatic fibrosis and decreased liver regeneration (Ferreira-Gonzalez et al., 2018). In this study, inhibition of TGF-β1 reduced biliary SASP secretion and increased hepatocyte proliferation and liver function (Ferreira-Gonzalez et al., 2018). Kasai portoenterostomy may resolve some features of cholestasis and fibrosis, but in a select group of patients’ fibrosis can remain following surgery (Kerola et al., 2019). In biliary atresia, TGF-β1 and decorin expression was elevated in lobular hepatocytes and fibrotic areas and correlated with liver fibrosis (Kerola et al., 2019). At 3-year follow-up, successful Kasai portoenterostomy significantly reduced hepatic TGF-β1 and connective tissue growth factor (CTGF) expression, while TGF-β2 expression was found to be increased (Kerola et al., 2019), signifying a central role for the TGF-β superfamily in promoting continued liver fibrosis after Kasai portoenterostomy. Considering that CTGF drives senescence in fibroblasts (Capparelli et al., 2012) it is reasonable to expect cholangiocyte SASP paracrine communication as central to biliary atresia progression and successfulness of Kasai portoenterostomy. Moreover, recent work found elevated levels of long non-coding RNA (lnc) H19 in serum and hepatic lysates from biliary atresia patients compared to healthy controls (Xiao et al., 2019). These H19 levels positively correlated with fibrosis in patients, marking it as a potential biomarker in disease detection. The authors also found elevated expression of fibrogenic markers, TGF-β, α-smooth muscle actin (αSMA) and ciliary localization (Cil)-1a, along with CK-7 (bipotential marker in HPC) in biliary atresia patients (Xiao et al., 2019). The isolated serum exosome analysis from biliary atresia patients revealed elevated lncH19, high mobility group AT-Hook 2 (HMGA2) and sphingosine-1-phosphate receptor 2 (S1PR2) compared to healthy controls, further implicating that hepatic fibrosis in biliary atresia could be paracrine in nature. It is plausible that these exosomes are cholangiocyte-derived and contribute to biliary atresia-associated fibrosis *via* paracrine crosstalk. While the discussed factors are not obligate SASP components, it is known that senescent and SASP cholangiocytes have an increased secretory component and could thus be a source of these pro-fibrogenic exosomes.A previous study found that *ex vivo* ductal organoids damaged with acetaminophen had induced caspase-3, apoptosis marker, and SASP factors expression, including TGF-β1, PDGF, IL-1β, IL-6, and TNF-α (Chusilp et al., 2020). This study indicates that cholangiocyte damage drives fibrotic response during biliary atresia. While fibrosis has been well established as a characteristic of biliary atresia, the crosstalk between biliary senescence and excessive fibrosis has yet to be captured. Understanding the profibrotic and prosenescent bile ducts and HPCs in biliary atresia may serve as a therapeutic target for disease attenuation in patients waiting for liver transplantation.

## Senescence in Fatty Liver Diseases

### Non-Alcoholic Fatty Liver Disease (NAFLD)/Non-Alcoholic Steatohepatitis (NASH)

Senescence has been the focus of recent studies with respect to various metabolic conditions including NAFLD (Zhou et al., 2021). Senescence, independent of aging, can exacerbate disease phenotypes resulting in the progression of the metabolic condition. NAFLD, or the more recent term MAFLD (metabolism-associated fatty liver disease) is the direct hepatic pathological manifestation of excess lipids and fats, primarily sourced from diet (Mantovani and Dalbeni, 2020; Younossi et al., 2021). Initiation of benign steatosis escalates into infiltration of leukocytes, mast cells and development of peribiliary fibrosis with this “second hit” (Zhu et al., 2020). This phenomenon can advance to non-alcoholic steatohepatitis (NASH) and, upon persistent insults, the liver progresses to cirrhosis and potentially hepatocellular carcinoma (HCC) which warrant liver transplantation.

The role of hepatocytes has been well studied in NAFLD (Venkatesh et al., 2017; Simon et al., 2020); however, there has been increasing interest in the contribution from cholangiocytes (Mendez-Sanchez et al., 2007; Kennedy et al., 2021; Zhou et al., 2021). A study showing evidence of DR in NAFLD was performed in rats fed with choline deficient high trans-fat diet where severe hepatic injuries were identified in areas of liver section with more CK-19 positive DR as identified by immunohistochemistry (de Lima et al., 2008). Cholangiocyte damage increases with hepatic steatosis development in NAFLD and NASH patients, further indicating cholangiocyte contribution to disease development (Natarajan et al., 2014). These early studies postulate a role from cholangiocytes in NAFLD and NASH, but true contribution is still be investigated. Cholangiocytes respond to fatty acid-induced lipo-toxicity by assuming a lipo-apoptotic phenotype *in vitro* (Natarajan et al., 2014; Natarajan et al., 2017) emphasizing a critical role for bile ducts in the phenotypic manifestation of NAFLD and describing a possible mechanism contributing to DR. It was also shown that NASH patients have increased DR and bridging fibrosis, which are two main hallmarks of cholangiocyte damage widely studied in cholangiopathies (Sorrentino et al., 2005). NAFLD and end-stage NASH patients also have increased DR and senescence (Kennedy et al., 2018b). Indeed, biliary senescence may be a significant contributor to NAFLD and NASH progression, similar to the cholangiopathies discussed above.

Similar to traditional cholangiopathies mast cells have been implicated in NAFLD/NASH progression. In histidine decarboxylase knock-out (HDC KO) mice, that have loss of endogenous histamine signaling, subjected to high-fat diet (HFD) there was reduced cholangiocyte damage and senescence, indicating a role for histamine signaling in bile duct damage during NAFLD progression (Kennedy et al., 2018b). Histamine has been previously indicated to aid in regulation of food intake and body weight *via* modulation of leptin signaling (Yoshimatsu et al., 1999; Jørgensen et al., 2007). Thus, this study crucially emphasizes the contribution of senescent cholangiocyte-mediated histamine release in fatty liver disease progression (Kennedy et al., 2018b). These studies define a crucial role for histamine in worsening liver phenotypes in NAFLD and NASH, but few studies have described mast cells and their derived components. NASH patients have increased serum insulin growth factor-1 (IGF-1, SASP factor) compared to healthy controls and C57BL6J mice fed western diet (WD) secrete enhanced IGF-1, specifically from cholangiocytes (Kennedy et al., 2021). Importantly, *in vitro* studies demonstrated that inhibition of mast cell IGF-1 receptor *via* antagonist treatment decreased migration toward damaged cholangiocytes. Further, this work demonstrates that expression of SASP factors from senescent cholangiocytes induce mast cell migration that promotes microvesicular steatosis *via* microRNA 144-3p (miR-144-3p)/aldehyde hydrogenase 1A3 (ALDH1A3) signaling (Kennedy et al., 2021). In general, this work shows that senescent cholangiocytes may worsen NAFLD and NASH *via* the recruitment of immune cells, including mast cells. In an analysis of 1,022 NAFLD patient biopsies, microvesicular steatosis has been identified as an advanced phenotype and strongly, positively correlation with hepatic ballooning, higher NAFLD activity score (NAS) and advanced fibrosis (Tandra et al., 2011). NAFLD patients also exhibit elevated serum histamine, implicating a role for mast cell-histamine in micro-vesicular steatosis development. It can be surmised from these studies that cholangiocytes exhibit a dynamic response to the damaging stimuli in NAFLD/NASH and secrete factors that increase mast cell infiltration that perturb injury. Interestingly, HFD fed older mice (8 and 18 months) showed increased M1 macrophage (proinflammatory) infiltration in liver indicating the effect of cellular senescence in immune cell infiltration during NAFLD (Fontana et al., 2013). As well, this work describes the role of aging in cellular senescence, but more studies to fully understand the mechanism is warranted. Further characterization of cholangiocyte sub-population (small/large) in HFD/WD models is required to understand how biliary epithelia contribute to the damage caused during NAFLD/NASH and may also highlight important information on biliary heterogeneity in terms of senescence induction that may help to understand cholestatic disease processes better.

### Alcoholic Liver Disease (ALD)

Liver pathophysiology associated with ALD is similar to NAFLD and includes severe steatosis, portal inflammation, and fibrosis ultimately leading to cirrhosis and liver failure (Yeh and Brunt, 2014). Although ALD can be differentiated from NAFLD based on etiology, aging is still an independent driving factor for the former. Previous work has shown that age was an influencing factor on the severity of liver injury, inflammation and fibrosis in mice fed with short- and long-term alcohol treatments (Ramirez et al., 2017). Further, the expression of SIRT1 was downregulated in aged animals and restoration of SIRT1 reversed the damage caused by binge alcohol feeding (Ramirez et al., 2017). The effects of alcohol on aging-related damage have been shown in Alzheimer’s disease, where alcohol directly reduced telomere length, one of the major manifestations of senescence (Yamaki et al., 2019). Based on these data one would assume that ALD affects cellular senescence through its cellular toxicity. As well, these studies implicate aging in the promotion of cellular senescence. The main enzyme responsible for elimination of alcohol in humans is ALDH2, and when mutated, this enzyme increases the risk of developing alcohol-related HCC in Asian population (Matsumoto et al., 2016; Chang et al., 2017). Hepatocyte specific *Aldh2* ^*−/−*^ mice subjected to 3-h ethanol gavage showed reduced aldehyde content compared to control, suggesting a role for other hepatic cells in hepatic metabolism and clearance of alcohol (Guillot et al., 2019). Interestingly, *in vitro*, ethanol provides a dose-dependent protection against cellular senescence by activating ALDH2 in endothelial cells, thereby suggesting a pivotal role of ALDH2 in senescence (Xue et al., 2019). ALDH2 mediates anti-senescence effects during chronic ethanol challenge by activation of SIRT1/p53 pathway in human aortic endothelial cells, *in vitro* (Xue et al., 2018). siRNA mediated knockdown of ALDH2 increased senescence-associated β-galactosidase, p21 and p53 expression in human vascular endothelial cells (Nannelli et al., 2018). Small molecule activator of ALDH2, N-(1,3-benzodioxol-5-ylmethyl)-2,6-dichlorobenzamide (Alda-1), alleviates the reduced aldehyde clearance and reverse hepatic steatosis in male C57BL6J mice subjected to 8-week alcohol treatment (Zhong et al., 2015). These studies identify an interesting role of ALDH2 in blocking cellular senescence, and this factor may be an important target to inhibit biliary senescence in ALD and other cholestatic disorders. Further, inhibition of ALDH3A1 by administration of synthetic inhibitors, tetraethyl thiuram disulfide (disulfiram), diethylamino benzaldehyde, or 4-amino-4-methyl-pent-2-ynthioic acid, S-methyl ester (ampalthiol ester), reduced cellular proliferation (Muzio et al., 2012). The reduced proliferation could be due to the inhibition of ALDH3A1 since its downstream effectors/targets CCL20, G-protein coupled receptor-37 (GPR37), and DEAD-box helicase 3 Y-linked protein (DDX3Y) have been implicated in growth and differentiation (Ruzinova and Benezra, 2003; Moreb et al., 2008). ALDH3A1-siRNA treatment in lung cancer cell lines, H522 and A549, reduced proliferation *via* increased peroxisome proliferation activated receptor (PPAR) expression (Oraldi et al., 2011). Thus, expression of ALDHs might be inversely correlated with onset and progression of cellular senescence.

Increased cellular senescence and miR-34a expression has been reported in the NIAAA murine model of ALD (Wan et al., 2017). Inhibition of miR-34a by Vivo-Morpholino reduced ALT, fibrosis, senescence and pathology score in ethanol-treated mice (Wan et al., 2017). Moreover, miR-34a has been implicated in the regulation of cellular senescence of cholangiocytes and hepatic stellate cells in ALD mice (Annable et al., 2015; Wan et al., 2015). Due to the increasing incidence of biliary senescence in other liver diseases, there may be an important role in for cholangiocyte senescence and SASP in ALD progression. As well, defining a set of miRs associated with biliary senescence could be key for delineating a senescence-associated miR profile or identifying therapeutic, prognostic or diagnostic targets.

Taken together, these studies highlight a role for aldehyde metabolism and miRNA signaling in mediating liver cellular senescence during ALD. The expression of ALDHs and miRNAs and their impact of biliary senescence on ALD is poorly understood. Further investigation is required to elucidate the role cholangiocytes, particularly senescent cholangiocytes, play in alcohol metabolism and damage progression in ALD.

## Future Directions and Conclusion

DR and biliary senescence/SASP are characteristic of cholangiopathies and are increasing in incidence during fatty liver disease progression and ALD, confirming biliary involvement, although mechanisms remain unclear. Biliary SASP factor secretion can increase the infiltration of mast cells and activate nearby cholangiocytes in cholestatic and NAFLD/NASH murine models, leading to increased inflammation and fibrosis. Mechanistic understanding of the role that biliary heterogeneity plays in SASP development has yet to be defined. Hepatocyte senescence drives hepatic steatosis in an age-dependent manner (Ogrodnik et al., 2017); however, further investigation into individual cell contribution to disease progression will provide novel therapeutic targets for patient treatment options. Future work should utilize cholangiocyte-specific mouse models to target biliary senescence and SASP factors to evaluate the role that these factors may play in the progression of cholangiopathies, NAFLD/NASH and ALD. As well, more studies are needed to define whether biliary senescence is an etiologic component of these liver disorders or a pathological consequence. Specifically in the context of NAFLD/NASH and ALD, that are not traditional cholangiopathies but nonetheless harbor cholestatic injury in a subset of patients, it is imperative to define which factors drive biliary damage in this setting. Understanding the mechanisms that promote biliary senescence in NAFLD/NASH and ALD is important since this injurious component can confer a worsening phenotype and poor prognosis. The recent onset of sophisticated experimental procedures, such as spatial transcriptomics and RNA sequencing, give researchers the capabilities to define the cellular niche during liver damage and can be utilized to describe biliary senescence in this setting. The power of RNA-sequencing can be used to evaluate the heterogeneity of biliary response to injury and induction of senescence. As well, spatial transcriptomics should be useful in defining the interaction of senescent cholangiocytes with the surrounding liver cells, and multiplex imaging assays can be used in a similar manner to define senescent cholangiocyte interaction with infiltrating immune cells. It is apparent that other experimental parameters are necessary to better define the onset of biliary senescence and the role that biliary senescence and SASP play during liver disease progression.

Biliary senescence and SASP will likely continue to gain attention in the field since cellular senescence/SASP factor secretion affects liver function and chronic liver disease progression (Meadows et al., 2019; Xiao et al., 2019; Kennedy et al., 2021). The use of senolytics may be a useful strategy to prevent senescence-associated damage in liver disease patients; however, this remains controversial. Recent work has shown that age-related NAFLD-HCC mouse models showed increased liver disease following senolytic treatment, indicating that senescence may be a consequence and not a driver of disease (Raffaele et al., 2021). Alternatively, chimeric antigen receptor (CAR) T cells can be engineered to specifically target senescent cells and were shown to ameliorate senescence-induced pathologies including hepatic fibrosis following carbon tetrachloride (CCl_4_) treatment (Amor et al., 2020). As described above, some preliminary work has been performed in animal models to evaluate the use of senolytics or other factors modulating biliary senescence as therapeutic agents for liver disease, but more work is necessary. Additional work in preclinical models should be carried out to further define the usefulness of inhibiting senescence and SASP in cholangiopathies, NAFLD/NASH and ALD. While senolytics are an intuitive pharmaceutical approach for the amelioration of liver damage associated with the above disorders, there are other indirect measures by which biliary senescence/SASP can be targeted. As described above, exosomes can be implicated in liver fibrosis progression in biliary atresia, but modulation of exosomes and the utilization of this signaling mechanism as a therapeutic need to be further investigated. Additionally, targeting age-related signaling mechanisms or miRs associated with biliary senescence can be an additional route to indirectly mediate this signaling component in liver disorders. A table of the key molecules discussed in this review that regulate biliary senescence during chronic liver diseases is listed in Table 1.

**TABLE 1:** Factors associated with cellular senescence in chronic liver disease.

Chronic liver disease	Cellular mechanism associated with senescence
Primary Biliary Cholangitis (PBC)	Telomere shortening, p16 and p21 expression
	Oxidative stress and ER stress
	Increased expression of CXCL11, CCL20, CCL2 and fractalkine
	Cholangiocyte autophagy including p62 signaling
	Loss of AE2 and the bicarbonate umbrella
	Enhanced secretin/SR signaling (early-stage PBC)
	Increased Bcl-xL (anti-apoptosis) expression
	Reduced Bmi1 (antioxidant) expression
Primary Sclerosing Cholangitis (PSC)	p16, p21, γH2A.X and SA-β-galactosidase expression
	Cholangiocyte secretion of SASP factors (IL-6, IL-8, CCL2, PAI-1)
	N-Ras activation in cholangiocytes
	Increased TGF-β1 signaling
	Upregulation of biliary secretin/SR signaling
	Mast cell-derived TGF-β1 and FXR signaling
	Cholangiocyte SCF secretion
	Increased biliary H2HR signaling
	Reduced biliary expression of FoxA2
	Increased age-related miRs and Twf-1 signaling
	Enhanced miR-34a/SIRT1 activity
	Increased Bcl-xL (anti-apoptosis) expression
Biliary Atresia	Reduced telomere length in hepatic tissues and peripheral leukocytes
	Enhanced biliary MHC I and II expression, and secretion of TNFα and TGF-β
	p16, p21 and NCAM expression
	Enhanced TGF-β1, TGF-β2, decorin and CTGF expression
	Increased serum and hepatic H19 levels
	Biliary expression of TGF-β, αSMA and Cil-1a
	Serum exosome H19, HMGA2 and S1PR2 levels
	Increased caspase-3, TGF-β1, PDGF, IL-1β, IL-6, and TNF-α expression in *ex vivo* ductal organoids
Non-Alcoholic Fatty Liver Disease (NAFLD)/Non-Alcoholic Steatohepatitis (NASH)	Dysregulated histamine/leptin signaling in cholangiocytes
	Biliary IGF-1 secretion and mast cell miR-144-3p/ALDH1A3 signaling
	Age-related M1 macrophage infiltration
Alcoholic Liver Disease (ALD)	Reduced SIRT1 activity
	Inhibition of ALDH2 and ALDH3A1
	Increased miR-34a

Table outlining the various signaling mechanisms and phenotypes associated with cellular senescence in chronic liver diseases, as discussed in the review. Senescence-associated factors and mechanisms are divided by disease, including PBC, PSC, NAFLD/NASH, and ALD.

**FIGURE 1:**
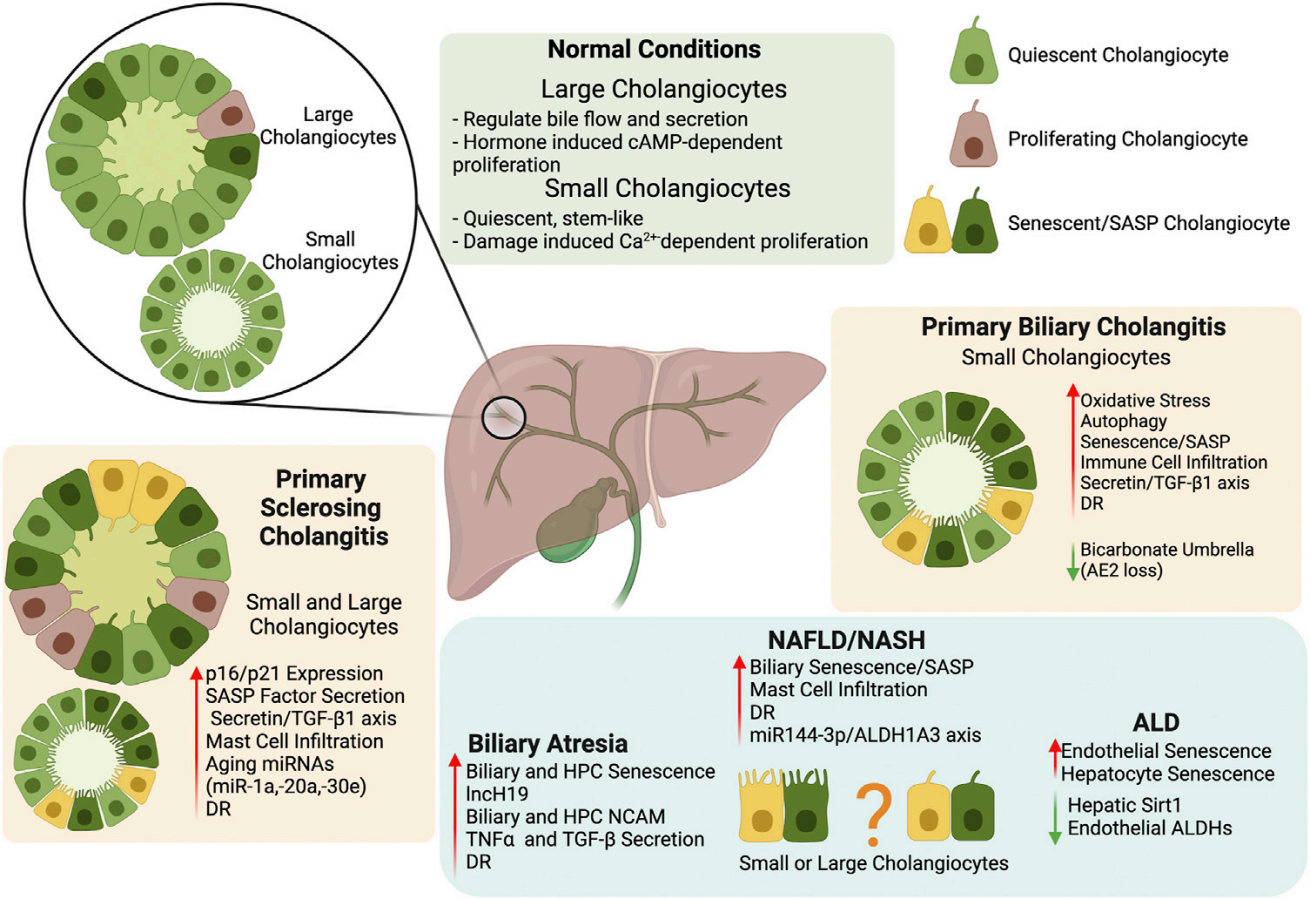
Biliary Senescence in Liver Disease. The key findings summarized in our review indicate that biliary heterogeneity and senescence/SASP factor secretion provide a novel direction for the study and treatment of chronic liver disease. Further exploration of the mechanism behind premature cholangiocyte senescence/SASP should help clarify the role of the biliary tree in chronic liver diseases. Created with biorender.com.

Due to the increase of senescence in cholangiopathies and fatty liver disease, targeting senescence and SASP secretion in cholangiocytes provides a novel direction in chronic liver disease treatment and a summary of critical findings described in this review can be found in Figure 1. Further investigation into the mechanism for premature biliary senescence/SASP factor secretion needs to be performed before this theory can be applied to clinical practice.

## Glossary

AE2: anion exchange protein 2
ALD: alcoholic liver disease
ALDH: aldehyde dehydrogenase
BA: bile acid
Bcl-xL: B cell lymphoma-extra-large
BDL: bile duct ligated/ligation
CAMK: calmodulin-dependent protein kinase
cAMP: cyclic adenosine monophosphate
CCL: C-C like chemokine ligand
CFTR: cystic fibrosis transmembrane conductance regulator
Cil1a: ciliary localization 1a
CREB: cAMP response element-binding protein
CTGF: connective tissue growth factor
CXCL11: C-X-C motif chemokine ligand 11
DDC: 3,5-diethoxycarbonyl-1,4-dihydrocollidine
DDX3Y: DEAD-box helicase Y-linked protein
DNA: deoxyribonucleic acid
DR: ductular reaction
ER: endoplasmic reticulum
ERK1/2: extracellular signal-regulated protein kinases-1/-2
FXR: farnesoid X receptor
GPR: g-protein coupled receptor
HFD: high fat diet
HPC: hepatic progenitor cells
IGF-1: insulin-like growth factor-1
IL: interleukin
MCP-1: monocyte chemoattractant protein 1
***Mdr2*** ^***−/−***^: multidrug resistant cassette 2 knock out
MEK: mitogen activated protein kinase kinase
MHC: major histocompatibility complex
miR: micro RNA
NAFLD: nonalcoholic fatty liver disease
NASH: nonalcoholic steatohepatitis
N-Ras: neuroblastoma RAS viral oncogene homolog
PBC: primary biliary cholangitis
PDFG: platelet derived growth factor
PKA: protein kinase A
PPAR: peroxisome proliferation activated receptor
pPKA: phospho-PKA
PSC: primary sclerosing cholangitis
SASP: senescence associated secretory phenotype
SCF: stem cell factor
SIRT1: sirtuin 1
αSMA: α—smooth muscle actin
SR: secretin receptor
Src: proto-oncogene tyrosine-protein kinase Src
TGF-β: transforming growth factor β
twf-1: twinfilin-1
UDCA: ursodeoxycholic acid
WD: western diet
